# Differences in the Effects of Saturated and Trans Fatty Acids on Metabolism and Anxiety‐Like Behavior in Mice

**DOI:** 10.1002/fsn3.71270

**Published:** 2025-11-24

**Authors:** Jin‐Cui Yang, Shuang Su, Ping Wang, Heng Zhang, Fan‐Zhi Kong

**Affiliations:** ^1^ Faculty of Medicine of University of Manitoba Joint Laboratory of Biological Psychiatry Mental Health Center of Shantou University, Shantou University Medical College Shantou Guangdong People's Republic of China; ^2^ The Fourth People's Hospital of Chengdu Chengdu Sichuan People's Republic of China; ^3^ Department of Medical Examination Center Mental Health Center of Shantou University Shantou Guangdong People's Republic of China

**Keywords:** 7‐nitroindole, anxiety, obesity, saturated fatty acids, trans‐fatty acids

## Abstract

High‐fat diets (HFDs) are linked to obesity, metabolic disorders, and increased anxiety and depression risks. However, the differential effects of saturated fatty acids (SFA) and trans‐fatty acids (TFA) on neuronal‐type nitric oxide synthase (nNOS) regulation of anxiety, metabolism, and mood remain unclear. This study aimed to compare the long‐term impacts of SFA and TFA on obesity, metabolism, and anxiety‐like behaviors, and to evaluate the role of nNOS in these processes. Mice were fed SFA and TFA diets for 12 weeks to induce obesity. Metabolic parameters such as body weight, blood glucose, blood lipids, liver enzymes, and body fat percentage were assessed. Anxiety‐like behaviors were evaluated using the Open Field and Sucrose Preference Tests in Weeks 11 and 12. The effect of 7‐NI (an nNOS inhibitor) on anxiety was also tested. Both diets induced obesity, glucose and lipid metabolism disorders, impaired liver function, and anxiety‐like behaviors. SFA resulted in greater weight gain, while TFA caused more severe blood glucose dysregulation and hepatic dysfunction. No significant difference in nNOS protein expression was observed between the groups. However, 7‐NI improved anxiety‐like behaviors in SFA‐fed but not TFA‐fed mice. SFA primarily caused greater physical obesity, whereas TFA led to more pronounced metabolic damage. The nNOS inhibitor 7‐NI reduced anxiety‐like behaviors in SFA‐induced obese mice but was ineffective in TFA‐induced obesity, highlighting distinct metabolic and behavioral effects between these two types of fatty acids.

## Graphical abstract

Saturated fatty acids (SFA) primarily caused greater physical obesity, whereas Trans‐fatty (TFA) led to more pronounced metabolic damage. Both SFA and TFA diets induced obesity and anxiety‐like behaviors. 7‐Nitroindole (7‐NI) improved anxiety‐like behaviors in SFA‐fed mice but not in TFA‐fed.

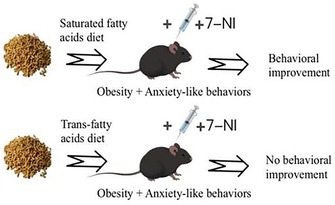

## 1 Introduction

Over 2.3 billion people are now overweight (Moschonis et al. 2022). It is predicted that by 2035, more than 4 billion people worldwide will be obese, representing over 51% of the world's population (Mozaffarian et al. 2006). Obesity is often associated with various complications (Chen et al. 2023; Caballero 2019; Lam et al. 2023), including an increased risk of various types of cancer, hypertension, cardiovascular disease, diabetes, and liver and kidney diseases (Moschonis et al. 2022; Piché et al. 2020; Blüher 2025). It also increases the risk of anxiety (Amiri and Behnezhad 2019) and depression (Henriques et al. 2025). Many dietary factors contribute to obesity, including excessive intake of saturated fatty acids (SFAs) and trans‐fatty acids (TFAs) (Tvrzicka et al. 2011; Ali Abd El‐Aal et al. 2019).

Saturated fat is a form of fat in the natural environment that is present in all fats and oils. Excessive intake of SFA has been proven to cause adverse health effects such as tissue inflammation, impaired insulin signaling, insulin resistance, and hepatic metabolism disorders (Li et al. 2022; Friedrich 2017; White 2009). TFAs are unsaturated fatty acids containing a trans structure and at least one double bond, which are not only recognized by the body, but are also extremely difficult to metabolize. TFAs come from two sources: natural TFAs found in small quantities (2%–6%) in meat from ruminants and in dairy products, and those produced through hydrogenation of polyunsaturated fats (Ali Abd El‐Aal et al. 2019; Brouwer et al. 2013; Martin et al. 2007). The ingestion of TFAs causes varying degrees of harm to human health (Li et al. 2020), increasing obesity, diabetes, fetal development issues, thrombosis, and cardiovascular risks (Souad 2024). The World Health Organization suggests that more than 278,000 deaths per year worldwide occur due to ingestion of industrial TFAs. In the United States, more than 30,000 deaths a year from coronary heart disease may be caused by trans fats in hydrogenated oils (Islam et al. 2019).

In the last few years, a growing number of studies, both in humans and in animals, have shown that the development of anxiety and depression is positively associated with long‐term SFA intake (Li et al. 2020; Gancheva et al. 2017; Qi and Gou 2024). In rodent studies, SFA‐rich diets impair spatial learning memory and induce anxiety‐ and depression‐like behaviors in mice (Ferreira et al. 2018). High‐fat intake causes metabolic disorders, which, in turn, increase the risk of depression and anxiety, and obesity is strongly associated with depression (Rihmer et al. 2008; Yu et al. 2023). 55% higher risk of depression in obese individuals than in the general population, whereas 58% higher risk of developing obesity in depressed individuals than in the general population (Luppino et al. 2010).

Neuronal‐type nitric oxide synthase (nNOS) is a signaling molecule downstream of the N‐methyl‐d‐aspartic acid receptor, which is widely distributed in several brain regions. nNOS is associated with mood disorders (Kourosh‐Arami et al. 2020; Zhu et al. 2023). The nNOS carboxy‐terminus contains a PDZ structural domain that interacts with proteins containing this structural domain. Notably, there is a PDZ structure in PSD‐95, and the interactions between PSD‐95 and nNOS are involved in the regulation of mood disorders (Qin et al. 2019; Cai et al. 2018). The 5‐hydroxytryptamine 2A receptor (5‐HT2AR) is the target of classical hallucinogens and is linked to depression through abnormal activity and expression levels (Adebo et al. 2025). Interestingly, nNOS exerts rapid antidepressant effects after being coupled to the 5‐HT transporter through the PDZ structural domain (Sun et al. 2022).

The nNOS inhibitor 7‐Nitroindole (7‐NI) is capable of stabilizing nNOS dimers and preventing nNOS degradation by the proteasome by decreasing the affinity between tetrahydrobiopterin and L‐arginine (Wang et al. 2022). The antidepressant and anxiolytic effects of 7‐NI have been studied in several animal studies for its antidepressant and anxiolytic effects (Yang et al. 2021; Kim et al. 2024). However, the effects of nNOS on anxiety in patients with obesity due to SFAs or TFAs remain unclear. Additionally, the differences in metabolism and mood disorders caused by long‐term intake of these fats require further study.

The objective of this study was to explore the differences in metabolic and mood changes occurring in obese mice with SFA and TFA diet‐induced obesity. Additionally, it was also explored the modulation of anxiety‐like behaviors by nNOS. We examined the relevant metabolic indices in mice fed SFA and TFAs over a 12‐week period, assessed their behavioral changes, and explored the differences in anxiety‐ and depression‐like behaviors induced by 7‐NI.

## 2 Materials and Methods

### 2.1 Animals and Diets

All animal operations were performed in accordance with the guidelines for animal research of the National Institutes of Health and approved by the Committee for the Management and Use of Laboratory Animals of Shantou University School of Medicine (Shantou, China; sumc2022–223). Seven‐week‐old C57BL/6N male mice (*n* = 42) were purchased from Zhejiang Vital River Laboratory Animal Technology Co. Mice were paired and housed in a well‐ventilated room with temperature maintained at 22°C–23°C, humidity at 50%–55%, and a 12‐h light/dark cycle. After 5 days of acclimatization, the animals were randomly divided into three groups and fed a customized diet. The diet was a granular that underwent natural air‐drying following extrusion through a pellet mill (Table 1; dietary components, Beijing Xiaoshu Youtai Biotechnology Co. Ltd., Beijing, China). The control diet group (CD, *n* = 8, AIN‐93G) was fed a normal diet; the SFA diet group (SFAD, *n* = 17, modified AIN‐93G) was fed a high‐fat SFA diet; and the TFA diet group (TFAD, *n* = 17, modified AIN‐93G) was fed a TFA diet (Table 2).

**TABLE 1:** Dietary components.

Dietary component	CD (g)	SFAD (g)	TFAD (g)	CD energy (kcal)	SFAD energy (kcal)	TFAD energy (kcal)
Casein	20	20	20	800	800	800
Cystine	0.3	0.3	0.3	12	12	12
Corn starch	45.22	7.275	7.275	1808.8	291	291
Maltodextrin	7.5	10	10	300	400	400
Sucrose	17.28	17.275	17.275	691.2	691	691
Dietary fiber	0.5	20.2	20.2	0	0	0
Lard	2	20.25	10.85	180	1823	977
Soybean oil	2.5	0	0	225	0	0
Hydrogenated soybean oil	0	0	9.4	0	0	846
AIN‐93G multi‐mineral	3.5	3.5	3.5	0	0	0
AIN‐93G multivitamin	1	1	1	40	40	40
Choline bitartrate	0.2	0.2	0.2	0	0	0
Total	100	100	100	4057	4057	4057

**TABLE 2:** Energy ratio.

Dietary component	CD	SFAD	TFAD
Carbohydrate, kcal%	70	35	35
Fat, kcal%	10	45	45
Carbohydrate, kcal%	20	20	20
Total energy, kcal/kg	4057	4057	4057

### 2.2 Study Design

**FIGURE 1:**
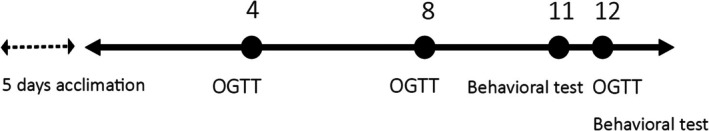
Experimental flow chart.

Body weight and fasting blood glucose levels were recorded weekly, and oral glucose tolerance tests (OGTT) were performed at Weeks 4, 8, and 12. Obese mice weighed at least 20% more than the average body weight of the control group were selected for the following experiments. Mice weighing less than the average weight of CD group were excluded from the behavioral test (Hariri and Thibault 2010). As a result, 3 mice in SFAD group and 3 mice in TFAD group were excluded. One in CD group was excluded for injury. Therefore, there were 7 mice in CD group, 14 mice in SFAD group, 14 mice in TFAD group. At the 11th week, they were subjected to the open field tests. At Week 12, sucrose preference tests were performed in the three groups. Then, seven mice were randomly selected from SFAD and TFAD groups for intraperitoneal injection of 7‐NI (30 mg/kg; Sigma, St. Louis, MO, USA). Before intraperitoneal injection, 7‐NI needs to be dissolved in DMSO in the following formulation: 15 mg of 7‐NI was dissolved in 0.5 mL of Dimethyl Sulfoxide (DMSO; Solarbio, Beijing) and titrated to 2 mL with normal saline The CD group was intraperitoneally injected with 1 mL/kg DMSO to rule out the possible effect of the solvent (Stanquini et al. 2018). Subsequently, five groups were gotten: (1) CD (*n* = 7); (2) SFAD (*n* = 7); (3) SFAD +7‐NI (*n* = 7); (4) TFAD (*n* = 7) and (5) TFAD +7‐NI (*n* = 7). Open field tests were performed 2 h after 7‐NI injection. Blood samples were obtained for liver enzymes (Alanine aminotransferase, ALT; Aspartate aminotransferase, AST) and lipid profiles (total cholesterol, triglycerides, and low‐ and high‐density lipoprotein levels) from mice in CD group, SFAD group, and TFAD groups that were not injected with 7‐NI. Finally, mice were euthanized by cervical dislocation, and then fat was stripped for fat weighing, while hippocampus and prefrontal lobe were stripped and stored at −80°C for subsequent Western blotting and immunoprecipitation experiments. Immunoblotting was performed to detect prefrontal PSD‐95 and nNOS expression levels. Immunoprecipitation was used to detect interactions between 5‐HT2AR, nNOS, PSD‐95, and nNOS (Figure 1).

### 2.3 Body Weight, Fasting Blood Glucose, and Oral Glucose Tolerance Test

After 12 h of fasting. Mice body weights were measured every week at 8:00 a.m. Blood was collected by tail clipping, and fasting blood glucose was measured using a glucometer at 30, 60, 90, and 120 min after 20% glucose gavage at Weeks 4, 8, and 12.

### 2.4 Behavioral Experiments

#### 2.4.1 Open Field Test

Mice were placed in the center of a rectangular box (50 × 50 × 40 cm), and their trajectories and time spent in the central area for 5 min were observed. The central zone is comprised of a square area 12.5 cm from the wall. The distance traveled and the amount of time spent in the central zone were used to assess the level of anxiety experienced.

#### 2.4.2 Sucrose Preference Test

Mice were continuously exposed for 48 h to two conventional water bottles placed in a cage, one is 1% sucrose water, the other is plain water. The amount of water in the bottle changed after 24 h. During the experiment, all mice were placed individually in cages after 12 h of fasting. We measured the volume of sucrose and drinking water consumed by the mice during the 12‐h dark cycle. Finally, we calculated the ratio of 12‐h sucrose consumption to drinking water consumption. A decrease in this ratio indicated the presence of pleasure deficit. Pleasure deficits are one of the three core symptoms of depression and can be used to assess depression‐like behaviors.

### 2.5 Body Fat

Body fat percentage was calculated as the percentage ratio of the abdominal fat pad to body weight, with the abdominal fat pad being the sum of retroperitoneal fat, perirenal fat, and epididymal fat (Zhou et al. 2009). Abdominal fat pads were separated, then weighed. The measurement of body fat was completed after conducting the behavioral experiment and obtaining serum in Week 12. Following euthanasia of the mice, the abdominal cavity of the mice was opened. Then the fat was placed on ice after being dissected from the peritoneum, epididymis, and perirenal areas. The tissues were weighed after a rinse with cold 0.9% saline solution and excess moisture being absorbed with filter paper. And then obtained final result through the body fat percentage formula.

### 2.6 Blood Lipid and Liver Enzymes Measurements

After completing behavioral studies in Week 12, blood was again collected from the mice. Plasma was isolated by allowing the blood to settle at room temperature, followed by centrifugation at 3000 r/min for 10 min. Finally, the separated plasma was tested for lipids and liver enzymes (Maccurate Bio technology Co. Ltd., Chengdu, CHINA; 7100 Automatic Biochemical Analyzer; Hitachi Ltd., Tokyo).

### 2.7 Western Blotting

Western blotting was used to detect nNOS and PSD‐95 protein levels. The tissue sample were diluted with a lysate prepared by mixing RIPA Buffer and PMSF at a 1:99 ratio. Then it was homogenized, and the protein concentration in the centrifuged supernatant was quantified using the Bicinchoninic Acid (BCA) Assay. A 10% sodium dodecyl sulfate‐polyacrylamide gel was employed for protein electrophoresis to achieve size‐based separation. And a volume of 15 μL protein lysate was loaded per well for gel electrophoresis, followed by transfer to a PVDF membrane. The PVDF membrane was soaked in a 5% skimmed milk powder solution for 60 min. Primary antibodies (PSD‐95/nNOS, 1:1000; Abcam, UK) were incubated overnight at 4°C. Membranes were then incubated with horseradish peroxidase‐coupled anti‐rabbit IgG secondary antibodies (1:2000; Beyotime, China) at room temperature for 1 h. Chemiluminescent reagents sufficiently covered the membrane, and the optical density values were analyzed using Image Lab software.

### 2.8 Immunoprecipitation

Twenty microliters of magnetic beads were aspirated and supplemented with 1 × TBS to a final volume of 500 μL. Magnetic separation was performed and repeated three times. The antibody working solution was prepared by mixing 1 × TBS with anti‐nNOS primary antibody at a 160:1 ratio and added to the magnetic beads, allowing sufficient binding. The supernatant was then removed. The protein solution was mixed with the beads and shaken at room temperature for 2 h. After magnetic separation and removal of the supernatant, 100 μL of 1 × sampling buffer was added, heated to 100°C for 5 min, and magnetic separation was repeated to collect the supernatant for immunoblotting.

### 2.9 Analysis

Statistical analyses were performed using SPSS 25.0, and GraphPad Prism 9 software was used for graphing. Normally distributed data were expressed as mean ± standard deviation, while non‐normally distributed data were expressed as median (interquartile spacing). Comparison of three or more groups of data, which were all normally distributed, was performed using one‐way ANOVA; otherwise, the Kruskal–Wallis test was used. Statistical results for weight, blood glucose, and glucose tolerance in this experiment were normal, and a multifactorial repeated‐measures ANOVA was used. Statistical significance was set at *p* < 0.05.

## 3 Results

### 3.1 Impact of *S*FAD *and* TFAD on Body Weight and Glucolipid Metabolism Disorders in Mice

**FIGURE 2:**
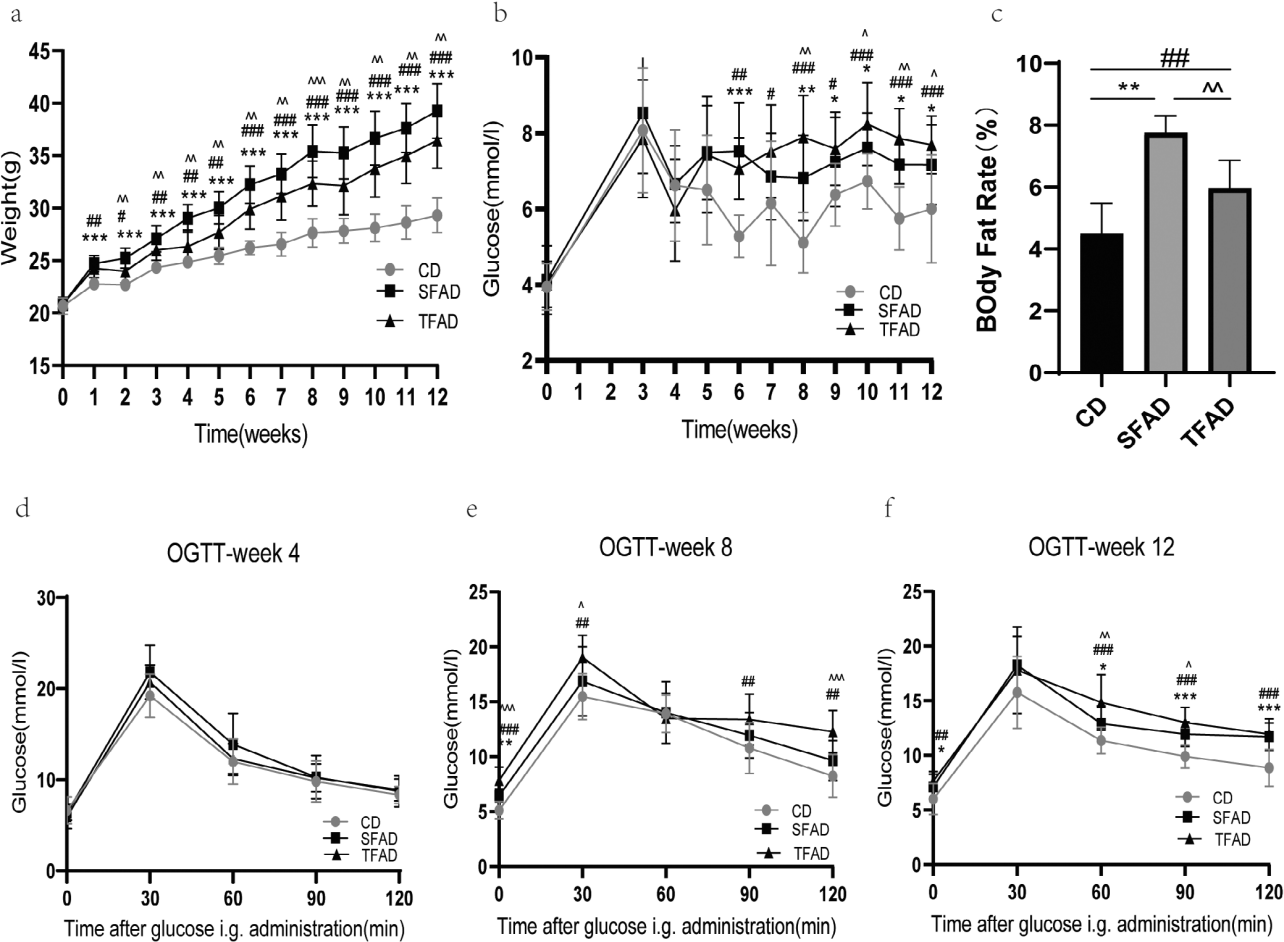
(a) Comparison of body weight in three groups at the same time and in the same group at different times. (b) Comparison of fasting blood glucose among three groups at the same time and in the same group at different times. (c) Comparison of body fat percentage between the three groups. (d–f) Comparison of blood glucose in the three groups at the same time and blood glucose comparison at different times in the same group. (a, c–f) Data analysis was performed using a repeated measures ANOVA method. (b) Data analysis was performed using the one‐way ANOVA. *p < 0.05, **p < 0.01, ***p < 0.001 (comparison between SFAD and CD groups); #*p* < 0.05, ##*p* < 0.01, ###*p* < 0.001 (comparison between TFAD and CD groups); ^*p* < 0.05, ^^*p* < 0.01, ^^^*p* < 0.001 (comparison between SFAD and TFAD groups).

Pre‐experiment, no significant differences were observed among groups in mouse weights (*p* = 0.774) and blood glucose levels (*p* = 0.881). From Week 1 to Week 12 of high‐fat diet (HFD) feeding, both SFAD and TFAD groups exhibited significantly higher body weights compared to the CD group (*p* < 0.05). Weeks 2–12, SFAD group body weights were significantly higher than TFAD group's (*p* < 0.05, Figure 2a). At Week 12, SFAD group's average mouse body weight increased by 34.02% vs. CD group, and TFAD group's by 24.97% vs. CD group. Additionally, fasting blood glucose levels increased significantly (*p* < 0.001) in both experimental groups compared to the CD group (Figure 2b). The results revealed a time‐dependent interaction between high‐fat diets and glycemic responses (Figure 2b; [F (2.39) = 3.47, *p* < 0.001]), which meant marked increase in fasting blood glucose following at least 5‐week feeding of high fat diets. Following 12 weeks of high‐fat diet (HFD) consumption, both the TFAD and SFAD groups exhibited significantly greater body fat percentages compared to the CD group (*p* < 0.01), with the SFAD group showing even higher levels than the TFAD group (*p* = 0.001) (Figure 2c).

Moreover, the results indicated that there was a time‐dependent interaction between high‐fat diets and glycemic clearance rate (Figure 2d–f; [F (2.39) = 3.916, *p* < 0.001]), which implied progressive deterioration of glucose metabolism in SFAD and TFAD group. At week 4, no significant differences were observed in OGTT outcomes across the three mouse groups (Figure 2d). By week 8, the TFAD group exhibited substantially elevated blood glucose levels compared to the other groups (*p* < 0.05) (Figure 2e). Glucose levels declined at 60 min in all groups, but at 90 and 120 min, the TFAD group showed significantly impaired glucose clearance versus the CD group. At Week 12, both SFAD and TFAD groups demonstrated markedly higher glycemic clearance rates than the CD group (Figure 2f). Between 60 and 90 min, the SFAD group had significantly poorer clearance than the TFAD group (*p* < 0.05), though no difference remained at 120 min. It suggested that the mice in the SFAD and TFAD groups exhibited more pronounced disruptions in blood glucose homeostasis compared to those in the CD group.

**FIGURE 3:**
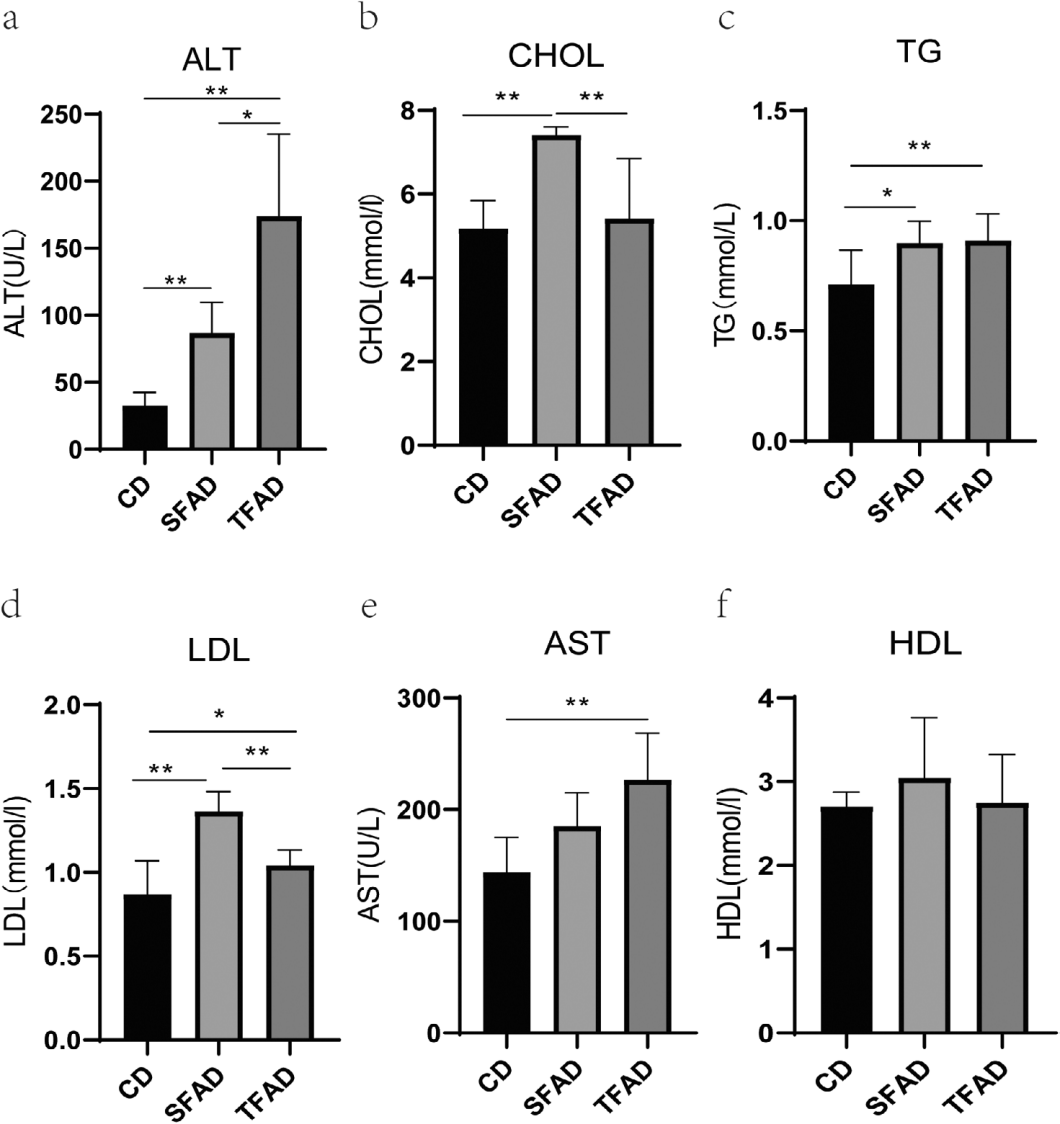
(a) Comparison of ALT levels, SFAD and CD groups, *p* < 0.01; TFAD and CD groups, *p* < 0.01; SFAD and TFAD groups, *p* < 0.05. (b) Comparison of CHOL, SFAD and CD groups, *p* < 0.01; SFAD and TFAD groups, *p* < 0.01. (c) Comparison of TG, SFAD and CD groups, *p* < 0.05; TFAD and CD, *p* < 0.01. (d) Comparison of LDL, SFAD and CD groups, *p* < 0.01; TFAD and CD, *p* < 0.05; SFAD and TFAD groups, *p* < 0.01. (e) Comparison of AST, TFAD and CD, *p* < 0.01. (f) Comparison of HDL, *p* > 0.05. (a–f) One‐way ANOVA. Comparison between the two groups under the horizontal line. *p < 0.05, **p < 0.01.

By Week 12, both SFAD and TFAD groups showed elevated serum ALT (Figure 3a), CHOL (Figure 3b), TG (Figure 3c), and LDL (Figure 3d) levels. AST levels (Figure 3e) were notably higher in TFAD than CD group (*p* = 0.005). HDL levels (Figure 3f) showed no significant differences across groups (*p* > 0.05), with SFAD group's LDL levels significantly exceeding those of TFAD group (*p* < 0.001).

### 3.2 SFA and TFAs Induced Short‐Term Anxiety‐Like and Depression‐Like Behaviors in Mice

**FIGURE 4:**
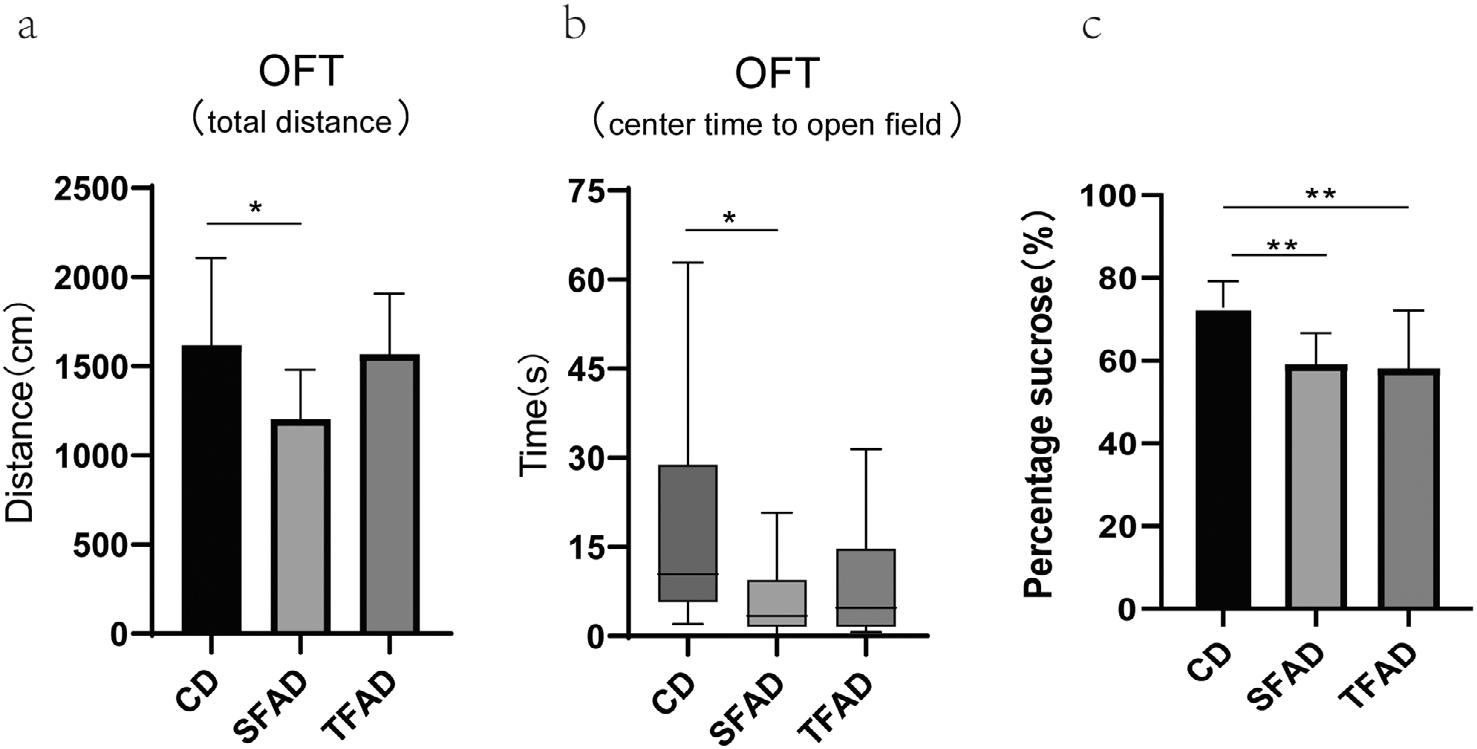
Obese mice fed HFD developed anxiety and depression‐like behaviors. Comparison between the two groups under the horizontal line. *p < 0.05, **p < 0.01.

To explore mood changes in obese mice, we conducted behavioral experiments. At Week 11, we performed open field experiments and found that the total distance traveled, which represents spontaneous locomotor ability, was significantly lower in the SFAD group than in the CD group (*p* = 0.016) and significantly lower in the SFAD group than in the TFAD group (*p* = 0.01). However, there was no significant difference between the CD and TFAD groups (*p* = 0.762) (Figure 4a). The central zone dwell time indicates the degree of anxiety, and less central zone dwell time was observed for the SFAD group than for the CD group (*p* = 0.046). No significant difference was observed between the central zone dwell times for the TFAD and CD groups (*p* = 0.149) (Figure 4b). Pleasure deficit is one of the core symptoms of depression. In this study, we used the sugar–water preference test to detect depression‐like behavior in obese mice. The results showed that the percentage of sugar–water preference of mice in both the SFAD and TFAD groups was significantly lower than that in the CD group (*p* < 0.01). In contrast, there was no significant difference between the TFAD and SFAD groups in terms of the percentage of sugar–water preference (*p* = 0.806) (Figure 4c).

### 3.3 Changes in Prefrontal PSD‐95 and nNOS Protein Expression Levels and Protein Interactions

**FIGURE 5:**
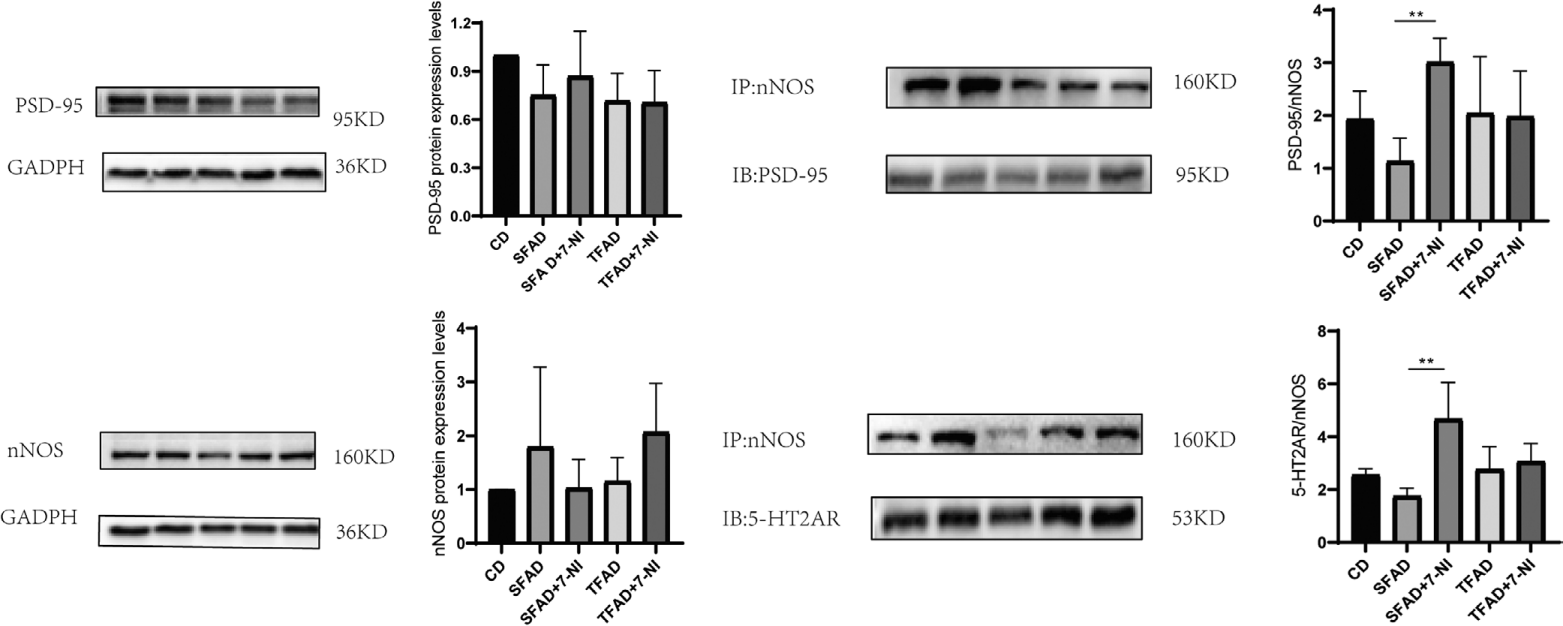
Changes in prefrontal PSD‐95 and nNOS protein expression levels and protein interactions. Comparison between SFAD AND SFAD‐7‐NI groups under the horizontal line: **p < 0.01.

There were no significant differences in PSD‐95 and nNOS protein levels in the prefrontal lobes between the groups. After intraperitoneal injection of 7‐NI, the SFAD PSD‐95/nNOS (*p* = 0.001) interaction was enhanced, as was the 5‐HT2AR/nNOS interaction (*p* = 0.009) (Figure 5).

### 3.4 Effect of 7‐NI on Anxiety‐Like Behavior in *HFD* ‐Induced Obese Mice

**FIGURE 6:**
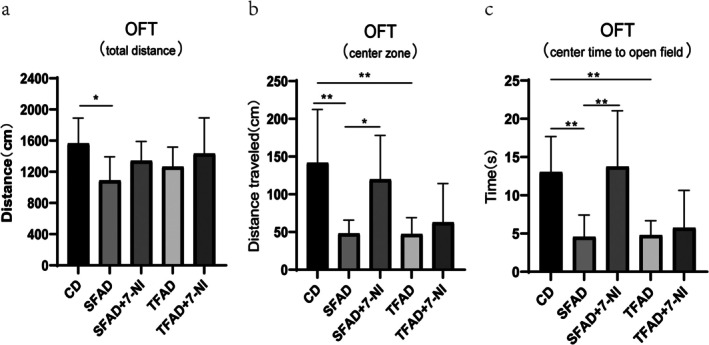
Effect of 7‐NI on anxiety‐like behavior in HFD‐induced obese mice (a) Comparison of total distance in five groups (b) Comparison of center zone distance in five groups (c) Comparison of center time in five groups. *p < 0.05, **p < 0.01.

The total distance traveled in the SFAD group was less than that in the CD group (*p* = 0.011), and there was no significant difference between the TFAD and CD groups (*p* = 0.097), indicating that the locomotor ability of mice in the SFAD group decreased (Figure 6a). Furthermore, there was no significant difference between the SFAD and SFAD +7‐NI groups or between the TFAD and TFAD +7‐NI groups (*p* > 0.05).

The central zone distance and central zone dwell time were used to test the anxiety‐like behavior of the mice. For central zone distance (Figure 6b), the SFAD group was significantly shorter than the CD group (*p* = 0.001) and the SFAD +7‐NI group (*p* = 0.01). The TFAD group also showed a significantly shorter distance than the CD group (*p* = 0.01), with no significant difference between the TFAD group and the TFAD +7‐NI group (*p* = 0.553). In terms of central zone dwell time (Figure 6c), both the SFAD (*p* = 0.002) and TFAD (*p* = 0.003) groups spent less time compared to the CD group, indicating anxiety‐like behavior. The SFAD +7‐NI group, however, showed a significant increase in central distance (*p* < 0.05) and dwell time (*p* = 0.01) compared to the SFAD group, while the TFAD and TFAD +7‐NI groups showed no significant difference (*p* = 0.708).

## 4 Discussion

In this study, after 12 weeks of HFD feeding, both the SFA and TFA groups of mice showed body weight gain, abdominal fat accumulation, liver enzymes, and disorders of glucose and lipid metabolism. Mice in the SFAD group exhibited higher body weight, abdominal fat accumulation, total cholesterol, and LDL levels compared to the TFAD group. However, the TFAD group showed more severe impairments in fasting blood glucose, oral glucose tolerance, and liver function. In conclusion, the SFAD was mainly characterized by obesity in terms of the appearance and accumulation of abdominal fat, whereas TFA‐induced obesity was less pronounced than that induced by the SFAD in mice; however, the metabolic damage in vivo was more severe.

A chronic HFD induced obesity in mice, as evidenced by weight gain and abdominal fat accumulation. This is consistent with the results of previous studies (Rosqvist et al. 2014). This may be because obesity is related to inflammation. It has been found that the knockdown of CD226‐stimulating molecules to inhibit macrophage M1 polarization reduces systemic inflammation, thereby reducing obesity (Ma et al. 2021). It has also been suggested that obesity is associated with rapid non‐apoptotic cleavage of caspase‐3 enzymes in astrocytes (Guyenet et al. 2013; Uddandrao et al. 2024). Furthermore, fatty acid‐induced leptin resistance is also involved in the diet‐induced obesity process (Engin 2024).

Glutamate transaminase and glutamate transaminase indices reflect liver function, and excessive intake of both SFAs and TFAs can cause liver function abnormalities. This may be linked to hepatic fat accumulation from excessive fatty acid intake (Zhang et al. 2023), resulting in hepatic cellular lipoatrophy and hypertrophy, oxidative stress, and inflammation (Robinson et al. 2014; García‐Berumen et al. 2022). In the present study, HFD‐fed mice showed abnormal lipid metabolism, as evidenced by elevated total cholesterol, triglyceride, and LDL levels. There were differences in lipid metabolism between the two fatty acid groups, as evidenced by a more significant elevation in total cholesterol and LDL levels in mice in the SFA group. A meta‐analysis showed a significant elevation in total cholesterol and LDL levels with regard to the intake of SFA in children and adolescents (Te Morenga and Montez 2017), which is consistent with the results of the present study. SFAs and TFAs reduce membrane fluidity and permeability, particularly in plasma membrane receptors involved in glucose and lipid metabolism. They also increase the expression of genes encoding adipogenic enzymes, leading to higher levels of serum triglycerides, abdominal fat accumulation, and insulin resistance (Cascio et al. 2012).

Both SFA‐ and TFA‐fed mice in this study had significantly elevated fasting blood glucose levels and impaired oral glucose tolerance. Oral glucose tolerance is a diagnostic criterion of diabetes. HFD‐induced insulin resistance may be associated with mitochondrial DNA damage, oxidative stress (Xia et al. 2024), adipose tissue inflammation (Lu et al. 2023), and impaired hypothalamic and leptin transduction pathways (Benoit et al. 2009; Liu et al. 2024). The results suggested that, compared to SFA group, TFA consumption led to more significant disruptions in fasting blood glucose levels and oral glucose tolerance. Discrepancies have been made regarding their impact on glucose homeostasis. Although it seemed association existed between TFA and all cause mortality, cardiovascular disease and coronary heart disease (CHD) (De Souza et al. 2015).

Both SFA‐ and TFA diet‐induced obese mice showed anxiety‐ and depression‐like behaviors in behavioral experiments. Several animal studies have shown that HFD‐induced obesity causes depressive behaviors (Yang et al. 2022). As regards clinical studies, a population‐based cohort study in 2022 confirmed that obesity is strongly associated with depression, with a higher body mass index showing a stronger relationship with depression‐related symptoms (Frank et al. 2022). The biological mechanisms behind obesity‐induced depressive comorbidities are not fully understood but may involve inflammation, neuroimmunity, the hypothalamic–pituitary–adrenal axis, and leptin regulation (Fulton et al. 2022; Milaneschi et al. 2019; Tzenios et al. 2023).

In a chronic variable stress model, long‐term intraperitoneal injection of 7‐NI ameliorated depression‐like behavior (Mutlu et al. 2015). However, in another study, 7‐NI failed to reduce amphetamine withdrawal‐induced depression‐like behavior in mice (Haj‐Mirzaian et al. 2018). Studies have also confirmed the antidepressant effect of 7‐NI in rats forced to swim, showing a significant reduction in immobility time during forced swimming tests. However, no effect was observed in the open field test (Yildiz et al. 2000), suggesting that while 7‐NI improves depression‐like behavior, it does not significantly affect anxiety‐like behavior. The differences in the results of these studies may be related to the methodology, study population, and duration of drug use. It has been found that 7‐NI reduces plasma cortisol concentration, decreases TNF‐α levels, and prevents oxidative stress and apoptosis (Almeida et al. 2015; Ferreira et al. 2012; Mohamed et al. 2013). These changes may be involved in the modulation of anxiety‐like and depression‐like behaviors by 7‐NI in rodents. In the present study, short‐term intraperitoneal injection of 7‐NI significantly improved anxiety‐like behavior in mice on a SFAD, but not in those on a TFAD. This suggests a differential effect of 7‐NI between the two types of fatty acids. To further investigate the reason for this difference, we examined the expression levels of prefrontal‐related proteins and found that 7‐NI had no significant effect on the expression levels of nNOS and PSD‐95 proteins in the short‐term. This may be attributed to the fact that the main effect of 7‐NI is to maintain the stability of the nNOS structure and prevent the degradation of nNOS, and that it does not alter the expression levels of nNOS proteins. Subsequently, we investigated whether protein interactions were involved in the regulation of anxiety‐like behaviors by 7‐NI, using immunoprecipitation to detect PSD‐95/nNOS and 5‐HT2AR/nNOS interactions in the prefrontal lobes of mice. The results revealed that 7‐NI significantly increased prefrontal PSD‐95/nNOS and 5‐HT2AR/nNOS interactions in mice in the SFAD group that were intraperitoneally injected with 7‐NI, whereas there were no significant differences in the levels of prefrontal PSD‐95/nNOS or 5‐HT2AR/nNOS interactions in the rest of the groups of mice. Since our previous results concluded that there are metabolic differences between mice in the SFA and TFA groups, it cannot be assumed that PSD‐95/nNOS and 5‐HT2AR/nNOS interactions are involved in the modulation of anxiety‐like behaviors in obese mice fed SFADs, and further validation is required.

This study has some limitations. Obesity causes various metabolic abnormalities, such as liver enzymes, blood lipids, and blood glucose. Whether these metabolic abnormalities influence the behavior of mice and the expression of proteins in the brain region needs to be further investigated. Moreover, no behavioral tests on cognitive function, memory, or behavioral despair were performed.

## 5 Conclusions

We found that chronic diets high in SFAs or TFAs induced obesity, liver enzyme abnormalities, and impaired glucose and lipid metabolism, leading to anxiety and depression‐like behaviors in mice. Compared to SFAs, TFAs are more detrimental to glucose regulation, insulin resistance, and liver enzyme disorders. Additionally, 7‐NI had an ameliorative effect on mood disorders in mice fed a SFAD, and there was a difference between SFAD‐ and TFAD‐induced mice in terms of the 7‐NI effect.

The number of obese people is increasing annually, and the prevalence of food processing is closely related to obesity. Long‐term intake of SFA can significantly contribute to obesity and glycolipid metabolic issues. TFAs have a more serious effect on liver glucose regulation, underscoring the importance of moderating saturated fat intake and reducing the intake of industrial TFAs. Moreover, obesity increases the risk of depression, suggesting that the biological mechanisms of depression should be studied from another perspective.

## Author Contributions

**Jin‐Cui Yang:** conceptualization (equal), methodology (equal), writing – original draft (equal). **Shuang Su:** data curation (equal). **Ping Wang:** conceptualization (equal). **Heng Zhang:** methodology (equal), resources (equal), writing – review and editing (equal). **Fan‐Zhi Kong:** funding acquisition (equal), writing – review and editing (equal).

## Funding

This work was funded by the Medical Scientific Research Foundation of Guangdong (A2022478).

## Conflicts of Interest

The authors declare no conflicts of interest.

## Data Availability

The data that support the findings of this study are available from the corresponding author upon reasonable request.
